# Bodyweight Changes Are Associated with Reduced Health Related Quality of Life: The Hordaland Health Study

**DOI:** 10.1371/journal.pone.0110173

**Published:** 2014-10-10

**Authors:** Gunhild Hervik Thorbjørnsen, Trond Riise, Jannike Øyen

**Affiliations:** 1 Department of Global Public Health and Primary Care, University of Bergen, Bergen, Norway; 2 Department of Rheumatology, Haukeland University Hospital, Bergen, Norway; Kyushu University Faculty of Medical Science, Japan

## Abstract

There is lack of studies investigating the association between bodyweight changes and health related quality of life (HRQL). The aim was to study the effect of relative changes in bodyweight over time on HRQL. In the Hordaland Health Study, 9276 men and 10433 women aged 40–47 years were included. Weight and height were measured and information on bodyweight changes during the last 5 years, physical activity and smoking was obtained from self–administered questionnaires including the Medical Outcomes Study MOS short form-12 including a Physical health Composite Score (PCS) and a Mental health Composite Score (MCS). Increasing bodyweight changes were associated with marked reduced scores in PCS and MCS also after adjustment for body mass index (BMI), physical activity and smoking. Men and women with a variation in weight with more than 15% during the last 5 years reported a mean score of MCS that was 0.48 standard deviation (SD) (3.9/8.1) and 0.35 SD (3.1/8.9) lower than those reporting a variation in weight less than 5%. No major differences were found between those who at date of examination were at the lower and higher end of the reported weight interval. There were no significant differences in the associations between men and women. Our findings confirm that increasing bodyweight changes are associated with reduced physical and mental health beyond what is related to BMI itself.

## Introduction

Weight gain along with increasing age in the population is a growing community problem according to The World Health Organization (WHO) [1]. In Norway, the average bodyweight has increased in the recent decades in line with decreased physical activity [2]. Overweight and obesity is strongly associated with a poor Health Related Quality of Life (HRQoL) [3]. HRQoL can be improved by regular physical activity [3]–[6], and weight loss as well as maintaining of weight loss has been suggested as being part of this, especially in combination with a restricted diet [5], [7]–[9]. A weight loss of 5–10% has been proposed to be enough for achieving health benefits [10], and improving HRQoL [11]. These recommendations have been based on studies that have investigated the effect of intervention related weight loss on HRQoL [12]–[14].

A recent large prospective study of female nurses showed a marked decrease in scores of physical components of HRQoL related to weight increase, a modest increase of the scores related to weight loss, and little association with weight changes related to mental health [15].

Using a general population including men and women aged 40–49 years we wanted to study how common bodyweight changes occurring over time were associated with physical and mental HRQL; if the associations could be explained by other lifestyle factors, and whether the associations differed between men and women.

## Methods

### Ethics statements

The study was approved by the Regional Committee for Medical Research Ethics review, REC West. Each participant signed an informed consent form.

### Study population

The study subjects were participants of The Hordaland Health Study (HUSK) in Western Norway where examination were conducted during 1997–1999 [16]. A total of 29400 subjects born 1953–57 were invited and 8598 men and 9983 women participated, yielding a participation rate of 57% and 70% for men and women, respectively. In addition, 2291 men and 2558 women born 1950–51 who had participated in a previous survey in 1992–1993 [16] were invited. Participation rates in these groups were 73% and 81%, respectively yielding 3269 participants from this cohort. This gave a total study population of 21850 participants who at date of examination were aged 40–49 years. A total of 135 patients did not respond to any of the HRQoL questions; 1326 had one of the 12 questions unanswered and 680 had from 2 to 11 unanswered questions. All these 2141 participants with missing data regarding HRQL were excluded, giving a total of 9276 men and 10433 women for analysis.

### Measurements

Measurements included height and weight, carried out by a health professional, with participants wearing light clothing and no shoes. In addition, highest weight and lowest weight during the last 5 years were self-reported before the examination. BMI was calculated as weight in kilograms divided by the square of height in meters. WHO categories of BMI classification was used in the analysis: underweight BMI <18.5, normal weight 18.5–25, overweight 25–30 and obese >30. The bodyweight changes during the last 5 years were classified into 0–5%, 5–15% and >15% changes of weight in kg compared to current weight. Participants with a bodyweight change <5% was considered weight maintainers. Participants who were measured to have a weight that was more than 3 kilograms outside the self-reported bodyweight range were included in a missing category for weight changes. This consisted of 1714 (8.7%) participants who had failed to report one or both weight limits, 1195 (6.1%) who at examination weighted 3 or more kilograms above their reported upper limit and 68 (0.3%) participants who weighted 3 or more kilograms less than their reported lower limit.

To estimate whether the weight change represented basically an increase or decrease in weight, we also classified the change according whether the current weight was in the lower or upper part of the reported weight interval during the last 5 years. Participants who had a current weight less or equal the mean of the interval were considered current “weigh loosers”, and participants with current weight above the mean considered current “weight gainers”.

Measurement of HRQL included the validated Medical Outcomes Study MOS short form-12 (SF12) questionnaire [17]. The 12 questions in this questionnaire are summarized into a Physical Health Composite Score (PCS) and Mental Health Composite Score (MCS). The PCS includes primarily the health related domains physical functioning, role limitation due to physical problems and bodily pain, while the MCS includes primarily health related social functioning, role limitation due to emotional problems, and mental health. The domains general health perception and vitality load substantially on both scales. The scales are initially standardized to a mean of 50 and a standard deviation of 10 according to the US population [18].

Information of smoking status and physical activity were also obtained from self-administered questionnaires. Smoking was categorized as current smoking, former smoking and non-smoking. Physical activity was registered as the number of hours spent weekly on light (without sweating or being out of breath) and heavy (causing sweating and breathlessness). This was categorized into three levels: <1 hour/week, 1–3 hours/week and >3 hours/week. In the multivariate analyses, we combined the effect of light and heavy physical activity by adding the categories giving hard physical activity twice the weight of light physical activity. This gave a summary score ranging from the lowest score of 0 (no light physical activity and no hard physical activity) to the highest score of 9 (>3 hours/week hard physical activity and >3 hours/week light physical activity).

The participants also reported whether they had or had had the following diseases: myocardial infarction, stroke, angina, diabetes, asthma or multiple sclerosis. The data were also matched to the Norwegian Cancer Registry for registration of cancer and date of diagnosis. Lastly, the respondents also reported their highest achieved education, yielding 5 levels of education; elementary school, lower secondary school, upper secondary school, lower university or college degree and upper university degree.

### Statistical analyses

Generalized linear models were used to estimate the association between the bodyweight changes, BMI, physical activity and smoking and the PCS and MCS. The analyses were performed stratified by gender. Differences in the associations between men and women were tested by including the relevant interactions terms in the model and including both men and women.

Two sensitivity analyses were performed to evaluate a possible effect of i) presence of chronic disease and ii) socioeconomic status. We rerun the analysis excluding all persons who at examination reported to have had any of the reported diseases including cancer. We also rerun the analyses including educational level as a categorical variable.

Two tailed P values <0.05 were considered statistically significant. Analyses were performed using the statistical tool Statistics Package for Social Science (SPSS) 19 for Windows.

## Results

The mean BMI was 26.2 for men and 24.7 for women. A total of 62.1% of the male population were overweight or obese compared to 38.8% for women (Table 1). Among men 22.3% reported a weight change during the last 5 years of less than 5% compared to only 13.0% among women. Around 50% of both men and women had never smoked. The mean score of the PCS and MCS in this Norwegian population was for men 50.8 (SD 7.4) and 50.6 (SD 8.1), respectively. Corresponding means for women were 49.3 (SD 8.9) and 49.5 (SD 8.9), respectively.

**Table 1:** Characteristics of the study participants in the Hordaland Health Study. Mean Physical health Composite Score (PCS) and mean Mental health Composite Score (MCS) due to body mass index, bodyweight changes, physical activity and smoking in men and women.

		Men (N = 9276)			Women (N = 10433)		
	Values	N (%)	PCS	MCS	N (%)	PCS	MCS
Body mass index	≤18.5	39 (0.4)	48.0	50.2	230 (2.2)	49.0	47.8
	18.5–25	3471 (37.5)	51.4	50.2	6145 (59.0)	50.2	49.6
	25–30	4620 (49.9)	50.8	50.9	3014 (29.0)	48.8	49.5
	>30	1131 (12.2)	49.1	50.6	1021 (9.8)	45.6	49.1
	p-value		<0.001	<0.001		<0.001	0.017
Bodyweight changes last 5 years.	0–5%	2065 (22.3)	51.6	51.7	1358 (13.0)	50.9	51.0
	5–15%	5057 (54.5)	50.9	50.6	5646 (54.1)	49.8	49.5
	>15%	815 (8.8)	48.4	48.1	1791 (17.2)	46.6	47.8
	Missing	1339 (14.4)	50.5	50.6	1638 (15.7)	49.1	49.9
	p-value		<0.001	<0.001		<0.001	<0.001
Bodyweight decrease/increase last 5 years.	“Loosers”	2187 (27.6)	50.5	49.9	1854 (21.1)	49.3	48.8
	“Gainers”	5750 (72.4)	51.0	50.9	6941 (78.9)	49.3	49.5
	p-value		<0.001	<0.001		0.70	0.002
Hard physical activity (with sweating and being out of breath)	<1h/week	4890 (53.9)	50.0	50.1	5976 (59.4)	48.3	48.9
	1–2h/week	2605 (28.7)	51.5	51.0	3002 (29.8)	50.7	50.1
	≥3h/week	1576 (17.4)	52.1	51.6	1091 (10.8)	51.3	50.7
	p-value		<0.001	<0.001		<0.001	<0.001
Light physical activity (without sweating and being out of breath)	<1h/week	1944 (21.5)	49.5	49.5	1613 (15.8)	47.1	48.0
	1–2h/week	3352 (37.1)	50.9	50.5	3901 (38.1)	49.3	49.4
	≥3h/week	3750 (41.5)	51.3	51.2	4715 (46.1)	49.9	50.0
	p-value		<0.001	<0.001		<0.001	<0.001
Current smoking	No	3377 (36.5)	51.2	51.0	3847 (37.0)	50.0	50.2
	Yes	3257 (35.2)	49.9	49.6	3720 (35.8)	48.1	48.4
	Previous	2623 (28.3)	51.1	51.3	2838 (27.3)	49.9	49.8
	p-value		<0.001	<0.001		<0.001	<0.001

The total number between variables may vary due to different number of missing data.

A low score on the PCS was significantly associated with high bodyweight changes, high BMI, little physical activity and smoking among both men and women (Table 1). Also for MCS there were significant associations with all covariates although the differences were generally smaller than for PCS.

Almost twice as many women (17.2%) compared to men (8.8%) reported bodyweight changes during the last 5 years of more than 15%. These individuals had the lowest mean PCS and MCS scores for both men and women. Further, never-smoking men (36.5%) and women (37.0%) reported the best PCS and MCS compared to current smokers. Only small differences were seen between never-smokers and previous smokers (Table 1).

Only 28% of men and 21% of women had at date of examination a bodyweight that was lower than the mean of the reported weight interval and were categorized as weight “loosers”. They were relatively evenly distributed through the bodyweight change categories (data not shown). These participants did not show higher SF12 scores, but in fact slightly lower scores, particularly for the MCS (Table 1).

In multivariate general linear analyses separately for men and women, bodyweight changes, BMI, smoking and physical activity remained significantly associated with both PCS and MCS in both genders (Tables 2 and 3).

**Table 2:** Associations between bodyweight changes, body mass index, smoking, physical activity and Physical (PCS) and Mental health Composite Score (MCS) in general linear multivariate model for male study participants (N = 8857)^a^ in the Hordaland Health study.

		Physical Component Summary				Mental Component Summary			
		Crude Mean	Diff adjusted^b^	95% CI	Eta-squared^c^	CrudeMean	Diff adjusted^b^	95% CI	Eta-squared^c^
Body weight changes	0–5%	51.5	0	-	0.007	51.7	0	-	0.014
	5–15%	51.0	−0.5	−0.9, −0.1		50.5	−1.4	−1.8, −1.0	
	>15%	48.4	−2.5	−3.1, −1.9		48.0	−3.9	−4.6, −3.2	
	Missing	50.5	−0.3	−0.9, 0.1		50.5	−1.0	−1.7, −0.4	
Body weight changes	“Looser”	51.0	0	-	0.001	49.8	0	-	0.001
	“Gainers”	50.5	0.5	0.1, 0.8		50.9	0.6	0.2, 1.0	
Body mass index	<18.5	48.3	−2.7	−5.0, −0.4	0.006	50.8	1.2	−1.3, 3.8	0.004
	18.5–25	51.4	0	-		50.1	0	-	
	25–30	50.8	−0.5	−0.8, −0.1		50.9	1.0	0.6, 1.4	
	>30	49.0	−1.9	−2.3, −1.3		50.6	1.2	0.6, 1.7	
Smoking	Never	51.4	0	-	0.005	51.0	0	-	0.005
	Current	49.9	−1.1	−1.4, −0.7		49.9	−1.0	−1.4, −0.6	
	Previous	51.1	−0.1	−0.5, 0.3		51.3	0.4	−0.0, −0.8	
Physical activity^d^		-	3.2	2.7, 3.7	0.016		2.3	1.7, 2.9	0.007

a Only participants with valid values on BMI, smoking and physical activity were included.

b Differences in mean score adjusted for all other covariates.

c Relative effect size.

d Included as continuous variable in the model with estimate corresponding to a difference between minimum (no light and hard physical activity) and maximum (>3 hours of both light and hard physical activity).

**Table 3:** Associations between bodyweight changes, body mass index, smoking, physical activity and Physical (PCS) and Mental health Composite Score (MCS) in general linear multivariate model for female study participants (N = 9880)^a^ in the Hordaland Health study.

		Physical Component Summary				Mental Component Summary			
		Crude Mean	Diff adjusted^b^	95% CI	Eta-squared^c^	Crude Mean	Diff adjusted^b^	95% CI	Eta-squared^c^
Body weight changes	0–5%	51.0	0	-	0.011	51.1	0	-	0.009
	5–15%	49.8	−1.0	−1.5, −0.5		49.4	−1.7	−2.2, −1.1	
	>15%	46.6	−3.2	−3.8, −2.5		47.8	−3.1	−3.8, −2.5	
	Missing	49.1	−1.1	−1.7, −0.4		49.9	−1.0	−1.8, −0.2	
Body weight changes	“Looser”	49.2	0	-	0.000	48.8	0	-	0.000
	“Gainer”	49.4	0.1	−0.4, 0.6		49.5	0.3	−0.1, 0.8	
Body Mass index	<18.5	49.1	−0.5	−1.7, 0.6	0.015	47.9	−1.3	−2.5, −0.1	0.001
	18.5–25	50.2	0	-		49.6	0	-	
	25–30	48.8	−1.0	−1.3, −0.6		49.5	0.2	−0.2, 0.6	
	>30	45.5	−3.8	−4.4, −3.2		49.1	0.2	−0.4, 0.8	
Smoking	Never	50.1	0	-	0.007	50.2	0	-	0.004
	Current	48.1	−1.5	−1.9, −1.1		48.4	−1.3	−1.7, −0.9	
	Previous	49.9	−0.1	−0.5, 0.4		49.8	−0.3	−0.7, 0.2	
Physical activity^d^		-	5.0	4.4, 5.7	0.023	-	2.8	2.1, 3.4	0.007

a Only participants with valid values on BMI, smoking and physical activity were included.

b Differences in mean score adjusted for all other covariates.

c Relative effect size.

d Included as continuous variable in the model with estimate corresponding to a difference between minimum (no light and hard physical activity) and maximum (>3 hours of both light and hard physical activity).

Men and women with a variation in weight with more than 15% during the last 5 years reported a mean score of MCS that was 0.48 standard deviation (SD) (3.9/8.1) and 0.35 SD (3.1/8.9) lower than those reporting a variation in weight less than 5%. Physical activity was the variable with the highest effect size related to PCS for both men and women. Bodyweight changes showed the strongest effect size on MCS for both genders.

Male weight “gainers” showed slightly higher scores of both PCS and MCS than weight “loosers” after adjusting for the other variables, while no significant differences were found for women.

An interesting finding was that BMI levels >25 was associated with reduced PCS compared to those with BMI <25, but not for MCS. Further, after adjustment for bodyweight changes and the other covariates there was actually a slightly increased MCS for BMI >25, particularly for men (Tables 2 and 3).

**Figure 1:**
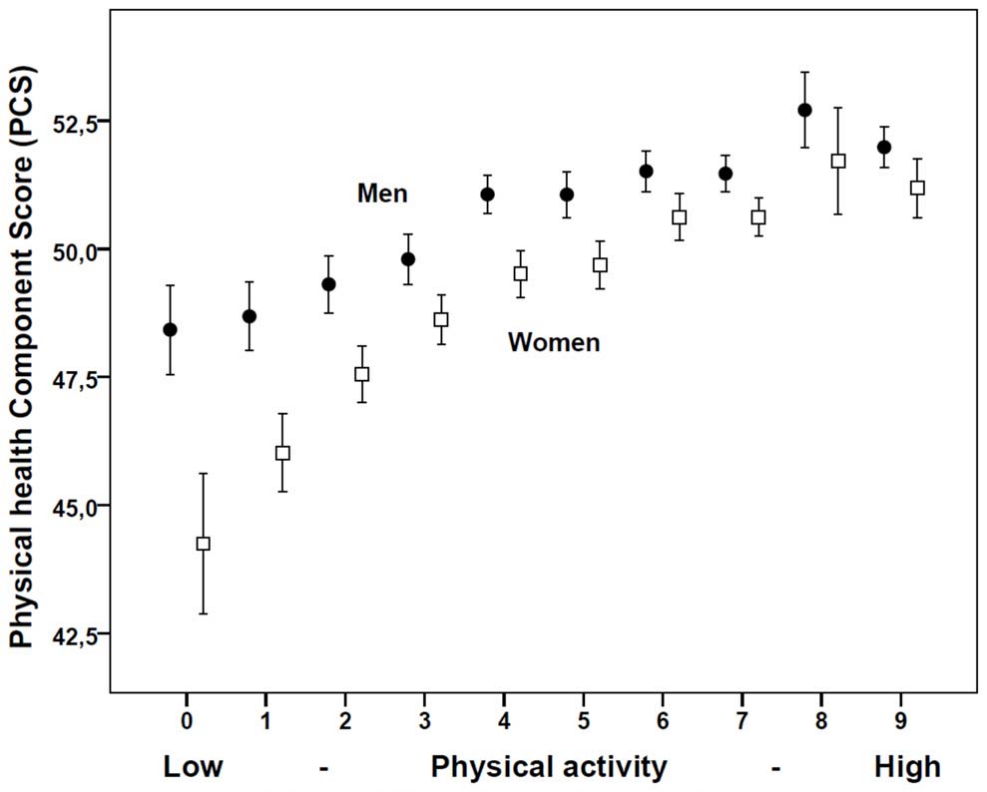
Physical health Composite Score (PCS)* by physical activity in men (N = 9276) and women (N = 10433) in the Hordaland Health Study. Physical activity is calculated as a weighted sum of hard and light physical activity with 3 corresponding to no light and hard physical activity and 12 corresponding to >3 hours/week of both light and hard physical activity. * Mean scores and 95% confidence interval based on raw scores. Similar effects were found after adjustment for confounding factors (body mass index, bodyweight changes and smoking).

**Figure 2:**
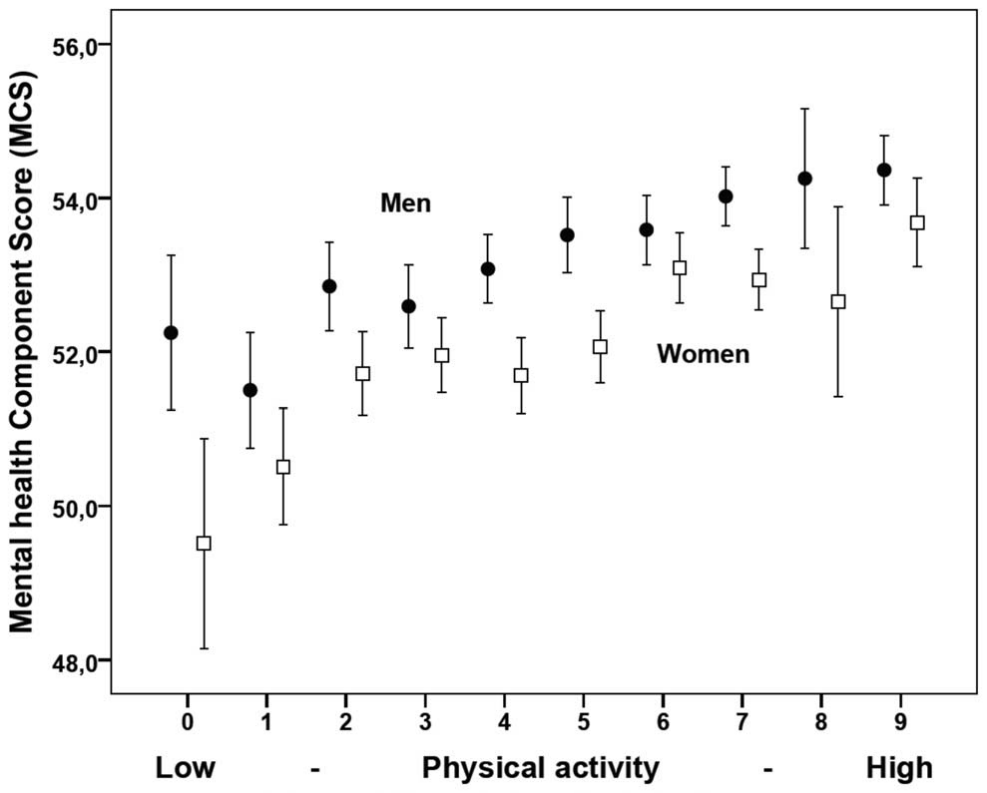
Mental health Composite Score (MCS)* by physical activity in men (N = 9276) and women (N = 10433) in the Hordaland Health Study. Physical activity is calculated as a weighted sum of hard and light physical activity with 3 corresponding to no light and hard physical activity and 12 corresponding to >3 hours/week of both light and hard physical activity. * Mean scores and 95% confidence interval based on raw scores. Similar effects were found after adjustment for confounding factors (body mass index, bodyweight changes and smoking).

We found a significant interaction between gender and physical activity on PCS, showing that the effect of physical functioning was stronger among women than among men (Figure 1). No significant interaction effects were found for MCS (Figure 2) and no significant difference in the effect between men and women were found for any of the other covariates.

The estimated R^2^ for the multivariate models including the main effects were 4.1% for PCS and 3.1% for MCS for men, while the estimates for women were 7.0% for PCS and 2.4% for MCS.

The reported frequencies of history of chronic diseases at examination were cancer 333 (1.7%), myocardial infarction 72 (0.4%), stroke 75 (0.4%), angina 103 (0.5%), diabetes 178 (0.9%), asthma 1163 (5.9%) and multiple sclerosis 65 (0.3%). Excluding these 1875 individuals (some had more than one disease) gave only minor changes of the estimates. The largest difference was found for body weight changes above 15% for men, where the decreased score was reduced from 2.5 to 1.9 when excluding those with chronic disease. The effect for this group on MCS (3.9) remained unchanged.

Repeating the multivariate models including educational level showed that the level of both PCS and MCS steadily decreased with decreasing level of education, with a difference of 3.4 and 2.8 in the PCS score between highest and lowest education for men and women, respectively. Similar but smaller differences were seen for MCS. Nevertheless, only negligible differences in effects of the other variables were seen. The largest difference was found for the estimate of body weight changes above 15% on PCS among women changing from 3.2 to 3.1 after adjusting for education.

To evaluate whether the exclusion of 2141 participants with missing values on the SF-12 questions likely have introduced any bias, we compared these participants with rest of the population (n = 19709) regarding age, sex, BMI and body weight changes. Among the 2141 participants who had not provided answer on all SF-12 questions there was a higher proportion of women compared to the rest of the population (62% vs 53%). There were no differences regarding age (mean 54.2 vs 54.3 years), BMI (mean 25.4 vs 25.4) or body weight changes.

## Discussion

In this study markedly reduced scores on physical and mental health was found among individuals who reported high bodyweight changes during the last 5 years. The effects remained after adjusting for current BMI, physical activity and smoking that all showed significant associations with the health scores. No major differences in scores were seen between those who at date of examination had a weight on the lower or upper end of the reported weight interval. Physical activity showed the strongest association with physical health, while bodyweight changes was the variable showing the strongest association with mental health.

In a previous large longitudinal study, weight gain in women was associated with reduced scores on 3 out of 7 self-reported health aspects including physical health. This was found irrespective of bodyweight at baseline [19]. An important question in that study is whether this negative effect was related to the weight gain itself or whether it was related to the high BMI that the participants had at end of follow-up. A systematic review of weight loss interventions studies found beneficial changes in HRQL following weight loss, regardless of method including or not including physical activity [20]. We adjusted for BMI at time of study and found that high weight fluctuations were associated with both reduced physical and mental health, regardless of current BMI.

We found that both men and women with low fluctuations in weight (0–5%) tended to have the best physical and mental health compared to individuals with larger fluctuations. Similar findings were observed in the longitudinal study of female nurses [15], although the relation between weight change with MCS were smaller in that study. The percentage of the variation of the HRQoL scores explained by all variables included was largest for the physical component also in the present study, but the relative importance of weight changes was strongest for the mental component, particularly for men. Only half as many men as women reported bodyweight changes of more than 15%, and this group of men could represent a particularly vulnerable group. The difference in the results between these two studies could also be related to the cutoff-point used for categorizing persons with high weight fluctuations. The study among the nurses used a cut-off of only 6.75 kg, while the present study used a percentage change of 15 that corresponded to a change of 12.6 kg for the average weighted man and 10.2 kg for the average weighted woman. Most participants in the present study had moderate fluctuations in weight (5–15%), which is in accordance with previously reported bodyweight gain due to age [19], [21], [22]. Unrealistic expectations of weight loss more than 15% might be a major reason for weight management failure and variations [23], as well as previous attempts to lose weight, which can be associated with current weight gain or greater variations [21], [24].

Further, in contrast to the study among the female nurses [15], we did not find that the participants who at examination were at the lower end of the reported weight interval scored better on the HRQoL scales compared to those on the higher end of the interval. The interpretation of this finding is difficult, but we observed the effect of natural variations in the general population that included persons who lost weight due to interventions and those who lost weight without intention. New studies need to distinguish between intentional and non-intentional weight loss when examining effects on health related outcomes.

HRQoL has previously been shown to be negatively associated with deviation from normal weight (BMI 18.5–25), particularly physical functioning [25]. We found that physical health was lower among overweighed and obese individuals as well as in underweighted men, which is in accordance with the study by Ford et al [25]. After adjustment, normal weighed tended surprisingly to have slightly poorer mental health compared to the other weight categories. This has also been observed previously particularly among men [26] but also among women [27]. Further, we found that underweighted women scored lowest on mental health. Underweight is commonly associated with poor physical health, disease and increased mortality [25], while overweight and obese individuals have a lower physical functioning in general [3]. Our results also showed that women were more physically and mentally affected by high BMI than men, which also has been reported earlier [28].

A marked association between physical activity and improved physical health is well known [6], [29], [30]. We observed an almost linear association between increasing physical activity and increasing physical and mental health that remained after adjustment for the other lifestyle factors including current BMI, suggesting that the beneficial effect of physical activity only to a limited extent is mediated through weight loss [31], [32]. Further, we also observed that women more than men seem to benefit from increased physical activity on physical functioning, a finding previously seen in relation to hard physical activity [33]. Increased physical activity have also previously been shown to improve HRQL after adjusting for weight changes, particularly among overweight women [34].

The main strength of this study is the large number of community-dwelling participants. However, the cross-sectional nature of the study precludes interpretations related to causality. Other limitations include the self-reported weight changes, since measured weight and self-reported weight perception are not necessarily coinciding [35]. Nevertheless, a good validity of self-reported weight has been found with a correlation coefficient of 0.96 between measured and self-reported weight [36].

Almost 10 percent of the participants had one or more missing items on the SF-12 questionnaire and could not be included in the analysis. However, these participants did not differ from the rest of the population regarding the included measures except for a skew gender distribution. Since we analyzed men and women separately, this suggests that no major bias has been introduced due to this missing category. Further, excluding participants with major chronic diseases did not substantially change the estimates, arguing against the associations found mainly reflecting an effect of diseases.

Our results show that reduced HRQL was markedly associated with large fluctuations in bodyweight, low levels of light and hard physical activity and current smoking. Improved mental health and especially physical health, was strongly and almost linearly associated with increased total physical activity, and women seem to benefit even more than men. Follow-up studies with better measures of weight change including a differentiation between intentional and non-intentional weight loss are needed to further determine how bodyweight changes are related to changes in HRQL.
